# Filling Teeth

**Published:** 1857-09

**Authors:** J. Taft


### DENTAL REGISTER OF THE WEST.

VOL. XI.—SEPTEMBER, 1857.—NO. 1.

### Original Essays and Communications.

### FILLING TEETH.

BY J. TAFT.

In the following general remarks upon filling teeth, the examination and treatment of the pathological conditions to which they are subject are left entirely out of view. The various steps of the operation will be described, and the different modes of accomplishing each step, at least so far as they possess any merit, will be examined. Our object now is to inquire how fillings can be most perfectly introduced into decayed teeth, under the circumstances in which we find them. We prefer, first, to consider that part of the subject which is merely operative or manipulative, and afterwards pathological conditions, their nature and treatment. By this course, but one principal subject is brought before the mind at one time. And we think it is better to take but one point, and consider it thoroughly, and pass on to another.

The operation of filling the teeth is an interesting and important one. It requires considerable peculiar talent and large experience for its perfect execution. It is the only means, at present in use, of effecting the object for which it is employed. The principal reliance for success is upon the operation. Therapeutic agents will do but little. So low is the organization, and so feeble the vital power, that far less is obtained from this source here, than in more highly organized structures. Nature does but little toward restoration or arrest. But such is the nature of the structure, that its decomposition is scarcely effected except by the active agency of potent foreign substances. The enamel is almost invulnerable to agents with which it ordinarily comes in contact.

The dentine is far more easily acted upon, and when there is a defect in the enamel, it is very liable to injury.

Scarcely any person arrives at mature age with a perfect set of teeth. Nine-tenths in our own country have decayed teeth at an early period of life. In operating for the preservation of any organ or set of organs so important and useful as the teeth, everything should be done to secure a successful result. No half-way work is admissible; perfect work should always be the aim. No one should rest contented until he has arrived at perfection, and none of our *best* operators have arrived at that point.

Every step in the process of filling a decayed tooth should be as perfect as possible, and the better method is to make each step perfect before passing to another.

I propose in the following remarks to analyze the subject somewhat, and examine the successive steps in the order in which they occur, endeavoring to ascertain the true method of accomplishing each.

EXAMINATION.

The first thing, when a case is presented, is an examination, and it should be thorough; for all the subsequent work will be modified by it. The points that should be noted in the examination are the following, viz:

The constitution and temperament of the patient.

The health.

The constitutional tendencies.

The general condition of the mouth.

The secretions, the saliva and mucus.

The mucous membrane and gums.

The constitution of the teeth, and their condition.

The number of teeth remaining in the mouth.

The number of teeth affected.

The extent of the decay, its nature, and the character of the agents producing it.

Thus the indications are ascertained. Whether general or local therapeutic treatment, if so, what kind, and to what extent; but to these particular reference will be made hereafter.

By the examination we ascertain how to proceed in the operation. If a great amount or but little labor is required, whether the operation will be a simple or a difficult one; and if difficult, what circumstances render it so, and, too, some conclusion is arrived at in regard to the permanency of the operation.

OPENING.

The next step is opening the cavity of decay, so that it may be approached and operated upon at all points. The particular manner of performing this is modified by the extent of the decay, and its position upon the tooth. In all cases the opening should be such as to give free access to all parts of the cavity, for removing effectually the decayed portion, for perfectly forming the cavity, and for introducing and thoroughly consolidating the filling. In central crown cavities of the molars and bicuspids, the projecting or pendant portions of enamel should be cut away.

There are cases, however, where such portion is firm and not liable to be broken, and where it can be well sustained by filling under, in which it is admissible to leave some projection. This is only true of those teeth that are of good, firm texture.

There are these two objections that may exist to these abrupt projections of enamel: It is very difficult, and in many cases impossible to fill perfectly beneath such portions; and again, it is very liable to be broken down during mastication.

For opening up these cavities, in many cases the bur drill alone will be quite sufficient, using them of different sizes, to open up the orifice gradually, so that too much violence may not be done to the teeth. In all very small cavities, the bur is all that is required.

In cases where the decay is more extensive, and the cavity larger, in connection with the drill, the chisel or heavy cutting instrument will be found very useful. By its use the operation will be very much facilitated, and less jarring will be communicated to the tooth ; but after it is used, the bur will be required to give a smooth, uniform border to the cavity. In these cases, the orifice should be almost or quite as large as the cavity is within.

In approximal decay, the cavity must be sufficiently exposed to enable the operator to see distinctly into it, and to manipulate freely in all parts of the operation. These cavities may be exposed by the use of the chisel, or the file, or by pressure. The separation of the teeth I have elsewhere considered at some length. (See Dental Register, vol. 8, p. 260.)

### REMOVAL OF DECAY.

After the cavity is opened, the next step in order is the removal of the decayed dentine. As a general rule, this should be entirely removed. There is, however, some variety of opinion upon this subject. This difference of opinion is in regard to cases where the entire or partial decomposition of the dentine has taken place quite to the pulp, and where, by its removal, the pulp would be exposed. It is maintained by some that decayed dentine affords a better protection to the nerve than any artificial covering can do, and hence it is better to let it remain. Its adaptation to the pulp is more perfect than any artificial covering would be, and it is not in every sense a foreign substance.

Upon the other hand, it is maintained that the decayed dentine will, being in an abnormal condition, irritate, and in many cases ultimately destroy the pulp. And again, that there is danger of making undue pressure upon the pulp, in filling on such softened portion.

In many cases, it is maintained that partially decomposed dentine will become dense again, if protected from the influence of foreign agents that decompose it. This sometimes would seem to be true. For in some cases where fillings have been introduced into cavities at the bottom of which a softened portion of dentine covered the nerve pulp, upon removing the filling in from one to five years afterward, the whole walls of the cavity were found to be equally and normally dense.

This, I suppose, would occur only in good constitutions, and under favorable circumstances; but with such a constitution and circumstances, where the softening was not too extensive, and the decomposition but partial, I should not hesitate to let it remain, and should entertain confidence of a happy result. This would certainly be better than to cut it all away and expose the nerve, and perhaps wound it, and then endeavor to cover it with some wholly foreign material that would not be perfectly adapted to it, that would press a little too hard at one point, and not touch at another, and that would be just as liable to be pressed down on the pulp as the softened dentine.

In this discussion, much depends upon the point whether partially decomposed dentine can retain its vitality. This I do not now propose to consider.

There are some particulars in regard to the removal of decay, about which there is no diversity of opinion. First, that all decomposed dentine should be removed from all parts of the cavity, where the pulp would not be exposed or injured thereby, and in *all* cases it should be entirely removed from the lateral walls of the cavity, and especially from the vicinity of the orifice. Even discolored dentine should be removed from this point.

Dentine often becomes changed in color when there is no apparent decomposition; such portion is usually, though not always, without vitality. It is not important to remove such changed portion, except for the appearance of the tooth; it will produce no change upon the living or normal part beyond it; and it is better material to be in contact with the living part than the metal of which the filling is made.

Decayed dentine is readily removed with the excavators, a description of which is given elsewhere. In any given case, that instrument should be selected that would be best adapted for the purpose, both in regard to size and the form of its edge, as well as curvature or inclination of its shaft. The edge of the instrument should come upon the walls of the cavity at such an angle as to accomplish the work most efficiently. It should be very sharp, and pressed firmly to the bottom of the decay at one side, and the principal part of it removed at one cut. With the proper instrument, and it in the right condition, all the decay should be removed from any cavity by a very few, firm, steady strokes. By this method the patient has less pain, and the work of the operator is facilitated. It is intolerable to think of being subjected to an awkward, clumsy hand, with a dull, ill-shaped excavator, scratching upon the surface of a decayed tooth, for, to the patient, an almost interminable length of time.

